# Abnormal Gene Expression Regulation Mechanism of Myeloid Cell Nuclear Differentiation Antigen in Lung Adenocarcinoma

**DOI:** 10.3390/biology11071047

**Published:** 2022-07-13

**Authors:** Zhongxiang Tang, Lili Wang, Ousman Bajinka, Guojun Wu, Yurong Tan

**Affiliations:** 1 Department of Medical Microbiology, School of Basic Medical Sciences, Central South University, Changsha 410078, China; tangzhongxiang@csu.edu.cn (Z.T.); wangll@csu.edu.cn (L.W.); obajinka@utg.edu.gm (O.B.); 2 China-Africa Research Centre of Infectious Diseases, School of Basic Medical Sciences, Central South University, Changsha 410078, China

**Keywords:** lung adenocarcinoma, myeloid cell nuclear differentiation antigen, gene methylation, SPI1, miRNA

## Abstract

**Simple Summary:**

Lung adenocarcinoma is the main pathological type of lung cancer with a very low 5-year survival rate. In the present study, through analyzing the mRNA, miRNA, and DNA methylation sequencing data from TCGA, combined with the downloaded clinical data, we accidentally found that both methylation and gene expression of some genes were up-regulated or down-regulated, which is in contradiction with our general understanding. To probe the mechanism, we selected MNDA associated with lung cancer prognosis for further analysis. The results showed that the imbalance of methylase and demethylase resulted in the demethylation of MNDA. Moreover, the expression of SPI1, the main transcription factor of MNDA, was down-regulated, while the two miRNAs hsa-miR-33a-5p and hsa-miR-33b-5p, which directly targeted MNDA, were up-regulated, thus inhibiting the expression of MNDA. In conclusion, the abnormal expression of MNDA in lung cancer is the result of the combined effects of transcriptional and post-transcriptional regulation.

**Abstract:**

Lung adenocarcinoma (LA) is the main pathological type of lung cancer with a very low 5-year survival rate. In the present study, after downloading the mRNA, miRNA, and DNA methylation sequencing data from TCGA, combined with the downloaded clinical data, comparative analysis, prognostic analysis, GO and KEGG analysis, GSEA analysis, methylation analysis, transcriptional regulation and post-transcriptional regulation were performed. We found that both methylation and gene expression of MNDA in LA were down-regulated, while high expression of MNDA was associated with good overall survival in LA. To probe the mechanism, further analysis showed that SPI1 was the main transcription factor of MNDA, but it was also down-regulated in LA. At the same time, the expression of eight target miRNAs of MNDA was significantly up-regulated, and the expression of hsa-miR-33a-5p and hsa-miR-33b-5p were verified to directly target MNDA. In conclusion, the abnormal expression of MNDA in LA is the result of the combined effects of transcriptional and post-transcriptional regulation.

## 1. Introduction

According to the latest report of the World Health Organization (WHO) and the International Agency for Cancer Research (IARC), lung cancer is the most common malignant tumor type in human beings, accounting for 11.6% of total cancer and 18.4% of total cancer deaths [1]. In lung cancer, non small cell lung cancer (NSCLC) is the most common, which is further divided into lung adenocarcinoma (LA) and squamous cell carcinoma. LA is the main pathological type of lung cancer, accounting for about 40% of the primary lung tumors and prone to brain metastasis [2,3]. Due to the lack of obvious early symptoms, the diagnosis of NSCLC is often in the late stage of cancer. Although the research on lung cancer has made great progress with technological advances, the pathogenic mechanism is very complicated, and the 5-year survival rate is only 16% [4]. Therefore, it is necessary to find better biomarkers for the diagnosis and treatment of lung cancer.

Myeloid cell nuclear differentiation antigen (MNDA) is a member of the immune-related hematopoietic IFN-inducible nuclear protein containing a 200-amino-acid repeat (HIN-200) family, which can act as a pattern recognition receptor to recognize foreign double-stranded DNA [5]. The N-terminus of MNDA is a highly helical pyrin domain (PYD). PYD belongs to one of the four subfamilies of the evolutionarily highly conserved death domain protein superfamily. The other three are death domains, death effector domains and caspase recruitment domain (CARD). These domains play important roles in innate immunity, inflammation, differentiation, apoptosis, and cancers through interacting to activate a variety of effector proteins, such as caspases and transcription factors [6]. The C-terminus of MNDA contains two adjacent oligonucleotide/oligosaccharide-binding (OB) domains, which are involved in nucleic acid recognition, and proteins with this structural feature are involved in DNA replication, recombination, repair and telomere maintenance and other processes [6]. Therefore, MNDA is mainly involved in autoimmune diseases by affecting the immune system [7]. However, the role of MNDA in LA is unclear and needs to be elucidated.

In recent years, epigenetic regulation has been increasingly considered to be an important goal of oncology [8]. Researchers have found that hypermethylation of tumor suppressors and hypomethylation of proto-oncogenes play important roles in the diagnosis and prognosis of tumors. DNA methylation involving the transfer of methyl to the C5 position of cytosine to form 5-methylcytidine, leading to gene inhibition or inhibition of the binding of transcription factors to DNA [9]. Abnormal DNA methylation can lead to the pathological process of organisms, carcinogenesis and development [10]. DNA methylation is often considered to be a marker of inhibiting gene transcription, by blocking the binding of transcription factors to gene promoter region in spatial structure. However, when we analyzed The Cancer Genome Atlas (TCGA) data, we accidentally found that both methylation and gene expression of some genes were up-regulated or down-regulated, which is in contradiction with our general understanding. This study attempted to explore whether this phenomenon is a special case or a sense of universality by means of bioinformatics, and describe the possible mechanism of this phenomenon.

## 2. Materials and Methods

### 2.1. TCGA Data Acquisition and Analysis

We used the University of California Santa Cruz (UCSC) xena (http://xena.ucsc.edu/; accessed on 20 February 2019) database to obtain the sequencing data and corresponding clinical information [11]. The UCSC database is derived from Level 3 data downloaded from the TCGA data coordination center. We downloaded the gene expression profiles of TCGA-LUAD containing 535 tumor samples and 59 normal samples, and TCGA-LUSC containing 502 tumor samples and 51 normal samples. Gene expression profiles were experimentally measured using the Illumina HiSeq 2000 RNA sequencing platform at the University of North Carolina (UNC) TCGA Center for genome characterization. Normalized counts for Fragments Per Kilobase of exon model per Million mapped fragments (FPKM) were performed on these datasets. The miRNA RNAseq data of TCGA-LUAD and the 450K methylated site data of TCGA-LUAD were also downloaded from UCSC xena. The miRNA RNAseq data including 450 tumors and 45 normal samples were normalized in TPM format. Additionally, the 450K methylated site data containing 460 tumor samples and 32 normal samples were measured experimentally using the Illumina Infinium HumanMethylation450 platform. The 450K integrated data and clinical information were downloaded from cBioPortal (http://www.cbioportal.org/; accessed on 20 February 2019). We used R language and limma package to analyze the difference between gene expression, miRNA expression and DNA methylation [11]. The differential mRNA was screened out according to the criteria of *p* < 0.05 and log2 ≥ |1|, and the differential miRNA was screened out according to the criteria of *p* < 0.05 and log2 ≥ |1|. The significant differentially different genes with methylated changes were screened under the conditions of *p* < 0.05 and | methylation change ≥ |0.2|. All of the above data were from the TCGA database, and their use and access were based on TCGA′s data access policy and publishing guidelines (https://cancergenome.nih.gov/publications/publicationguidelines; accessed on 20 February 2019). Therefore, no additional ethics committee approval was required.

### 2.2. Comparative Analysis of MNDA between LA and Normal Tissues

Paired and unpaired analytical methods were used to compare MNDA expression between LA and normal lung tissue. The UALCAN database was used to analyze the expression of MNDA protein (http://ualcan.path.uab.edu/; accessed on 6 May 2022) and the methylation changes at its promoter position in MNDA [12,13]. The Human Protein Atlas (HPA) (https://www.proteinatlas.org/; accessed on 6 May 2022) database was used to assess protein expression of MNDA by immunohistochemistry [14]. The relationship between the methylating site of MNDA and the expression of MNDA in 450K was analyzed.

### 2.3. Intersection of Differential mRNA and Differential Methylation

Intersection of up-regulated mRNA expression and methylation and down-regulated mRNA expression and methylation was obtained. Then, the genes for further study were screened under the conditions that genes with hypermethylation and high expression were related to good prognosis, or genes with low methylation and low expression were associated with good prognosis.

### 2.4. Oncomine Analysis of the Expression of MNDA

The expression of MNDA in published research was studied by using online database Oncomine [15] (https://www.oncomine.org/; accessed on 21 August 2019) under the condition of Gene: MNDA; Analysis Type: Lung Cancer vs. Normal Analysis; Data Type: mRNA and ORDER BY: Under-expression: Fold Change. 

### 2.5. Functional Analysis of MNDA in LA

The samples in LA were divided into the low expression and high expression groups according to the expression level of MNDA for differential gene analysis. *p* < 0.05 and log2 ≥ |1| were used as the screening conditions for significant differences. GSEA analysis was performed to identify pathways based on the obtained transcript sequences of TCGA [16,17]. Gene Ontology (GO) function and Kyoto Encyclopedia of Genes and Genomes (KEGG) pathway enrichment analysis were performed to analyze the function and pathways of MNDA-related DEGs. The ssGSEA algorithm in the GSVA package was used to evaluate the immune-infiltrating relationship of MNDA [18].

### 2.6. TF Chip-Seq Analysis of SPI1 Binding to the MNDA Promoter

The Encode (https://www.encodeproject.org/; accessed on 10 September 2019) database was used to analyze the binding of SPI1 to the MNDA promoter and visualized with the WashU Epigenome Browser online tool (http://epigenomegateway.wustl.edu/browser/; accessed on 10 September 2019) [19,20].

### 2.7. Prediction of MNDA Target miRNA by TargetScanHuman

The predictive target miRNAs of MNDA mRNA were selected using the online database TargetScanHuman (http://www.targetscan.org/vert_72/; accessed on 19 September 2019) [21].

### 2.8. Cell Culture and Real-Time RT-PCR

Human normal lung epithelial BEAS-2B cells and lung cancer cell lines PC9, H1299, and A549 cells were cultured in DMEM plus 10% fetal bovine serum (FBS) at 37 °C and 5% CO_2_. Total RNA was extracted from cells using TRIzol reagent (Takara, Kyoto, Japan). Primers were synthesized by Sangon Biotech Co., Ltd. (Shanghai, China), and the MNDA primers were 5′-ACTGACATCGGAAGCAAGAGGG-3′ (forward) and 5′-TGCAGATGTGCTGGCTCCTGAG-3′ (reverse), the SPI1 primers were 5′-GACACGGATCTATACCAACGCC-3′ (forward) and 5′-CCGTGAAGTTGTTCTCGGCGAA-3′ (reverse). Each sample was reverse-transcribed into cDNA using TransScript Uni All-in-One First-Strand cDNA Synthesis SuperMix for qPCR (Transgen, Tianjing, China). Then, the cDNA was synthesized by reverse transcription and amplified using 2× SYBR Green qPCR Master Mix (Bimake, Houston, TX, USA) according to the manufacturer’s instructions. qPCR was performed at 95 °C for 3 min, and 40 cycles of 95 °C for 15 s, 60 °C for 30 s, and 72 °C for 30 s. GAPDH and β-actin were the internal references for the target genes. The relative expression levels of mRNA were calculated using the 2−ΔΔCT method. 

### 2.9. Cell Transfection

The SPI1 siRNAs and the corresponding negative controls were synthesized by Ruibo Biotechnology (Guangzhou, China). 293T cells were seeded in 6-well plates for 24 h before transfection. Transfection was performed using LipofectamineTM 2000 (Invitrogen, Carlsbad, CA, USA) according to the manufacturer’s instructions. The siRNA and si-NC were diluted to a concentration of 100 nmol/L, and the subsequent treatment was performed at 24 h post-transfection. The transfection efficiency was detected using RT-qPCR. 

### 2.10. Binding of SPI1 with MNDA Promoter

Primers were designed to amplify the SPI1 binding site in the MNDA promoter region, and PCR products were purified by Qiagen PCR purification kit (Qiagen, Hilden, Germany) A total of 10^5^ cells were lysed with 1 mL of RIPA buffer (Dingguo, Beijing, China) containing protease inhibitors, incubated on ice for 30 min, and then centrifuged at 13,000× *g* for 10 min. The supernatants were collected, and quantification was performed using BCA kit. After 200 ng DNA and 100 μg protein in reaction buffer (50 mM Tris PH 7.5, 100 mM KCl, 10% glycerol, BSA 1 mg/mL, 1 mM β Beta-Mercaptoethanol) with a final volume 20 μL were incubated for 20 min at room temperature, all of the binding reactions were loaded onto 8% non-denaturing polyacrylamide gel and run at a voltage of 100 v for the desired distance. After the retarded bands were determined by ethidium bromide, the binding of SPI1 to the promoter was detected by incubation with PU.1/SPI1 rabbit primary antibody (ab230336, Abcam, Shanghai, China) and corresponding secondary antibody (Bioss, Beijing, China, 1:5000).

### 2.11. Dual-Luciferase Reporter Assay

The 3′-UTRs of MNDA containing hsa-miR-33a-5p, hsa-miR-33b-5p, hsa-miR-744-3p and hsa-miR-183-3p binding sites were amplified by Nanjing Genscript Biological Technology Co., Ltd (Nanjing, China)and cloned into the pmirGLO vector (Promega, Madison, Wisconsin, USA). Then, the wild type of MNDA or mutant MNDA 3′-UTRs were constructed. Then, the constructed plasmids were co-transfected with hsa-miR-33a-5p, hsa-miR-33b-5p, hsa-miR-744-3p, hsa-miR-183-3p mimics or mimic-NC into 293T cells using Lipofectamine 2000 (Invitrogen, Carlsbad, California, USA) according to the manufacturer’s instructions. After 48 h of transfection, luciferase activities were measured by the Dual-Luciferase Reporter System (Promega, Madison, WI, USA).

## 3. Data Analysis

The statistical analysis and figure construction were completed in R software version 3.6.3 (https://www.R-project.org/; accessed on 20 January 2019) using packages including Ggplot2 and Ggpubr. KM plot was performed and analyzed using a log-rank test. GraphPad Prism 8 was used for the calculation of qPCR results. Student′s *t* tests were used for differential expression analysis. Pearson correlation coefficients were used for correlation analysis. There was a significant difference if *p* value < 0.05.

## 4. Results

### 4.1. MNDA Is Down-Regulated in LA

**Figure 1:**
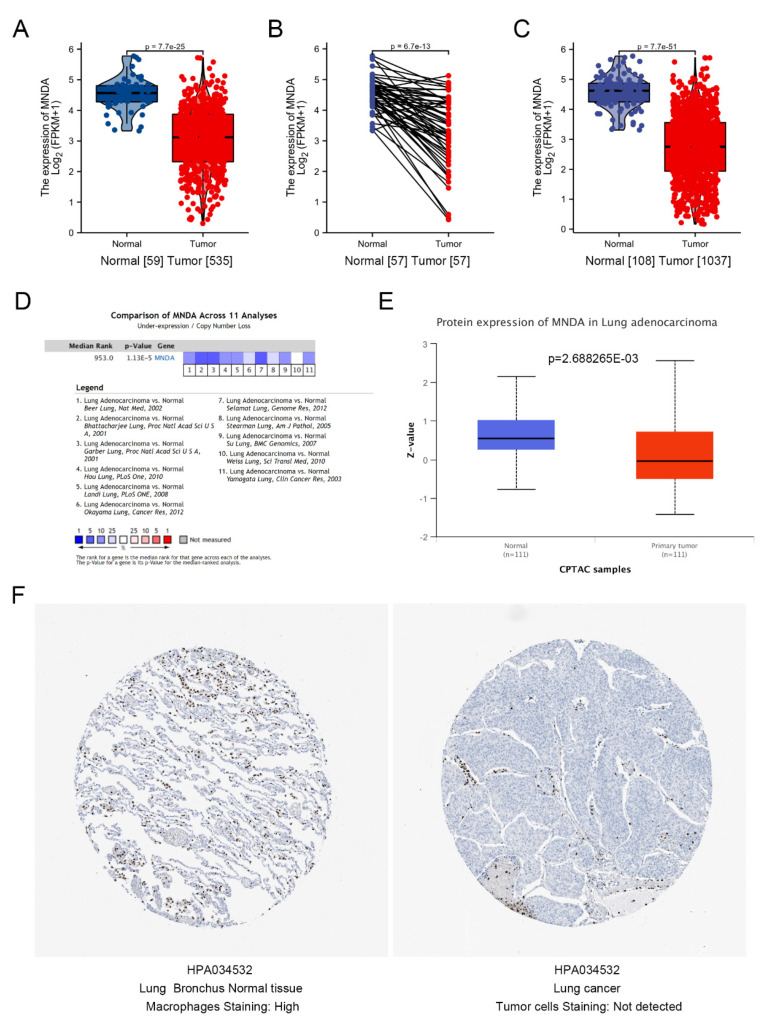
MNDA is down-regulated in LA. (**A**) Unpaired analysis showed that the mRNA expression level of MNDA in LA was significantly lower than that in normal tissues (Normal = 59, Tumor = 535, *p* = 7.7 × 10^−25^). (**B**) Paired analysis showed that the mRNA expression level of MNDA in LA was significantly lower than that in normal tissues (Normal = 57, Tumor = 57, *p* = 6.7 × 10^−13^). (**C**) Comparison of mRNA expression levels of MNDA in NSCLC and normal lung tissues (Normal = 108, Tumor = 1037, *p* = 7.7 × 10^−51^). (**D**) Comparative analysis of MNDA expression in 11 LA studies in the Oncomine database. (**E**) The protein expression of MNDA in LA from CPATC samples in the UALCAN database was analyzed. (**F**) Comparison of immunohistochemical staining for MNDA in LA and lung normal tissue from the HPA.

To evaluate the expression of MNDA in LA, we used paired and unpaired methods to analyze the expression of MNDA in TCGA, and the results showed that MNDA was significantly down-regulated in LA (Figure 1A,B) and also significantly down-regulated in NSCLC (Figure 1C). To determine if the decreased expression of MNDA in LA is a common phenomenon, we analyzed the Oncomine database and found that MNDA mRNA decreased in all 11 studies of LA (Median Rank = 953, *p* value = 1.13 × 10^−5^, Figure 1D). Our next analysis, using CPTAC in the UALCAN database, showed that the protein expression of MNDA in LA was also significantly lower than that in normal tissues (Figure 1E). This result was consistent with our analysis of the immunohistochemical results of MNDA protein expression in LA and normal lung tissue from Human ALTAS database (Figure 1F).

### 4.2. High Expression of MNDA Is Related to the Good Prognosis of LA

**Figure 2:**
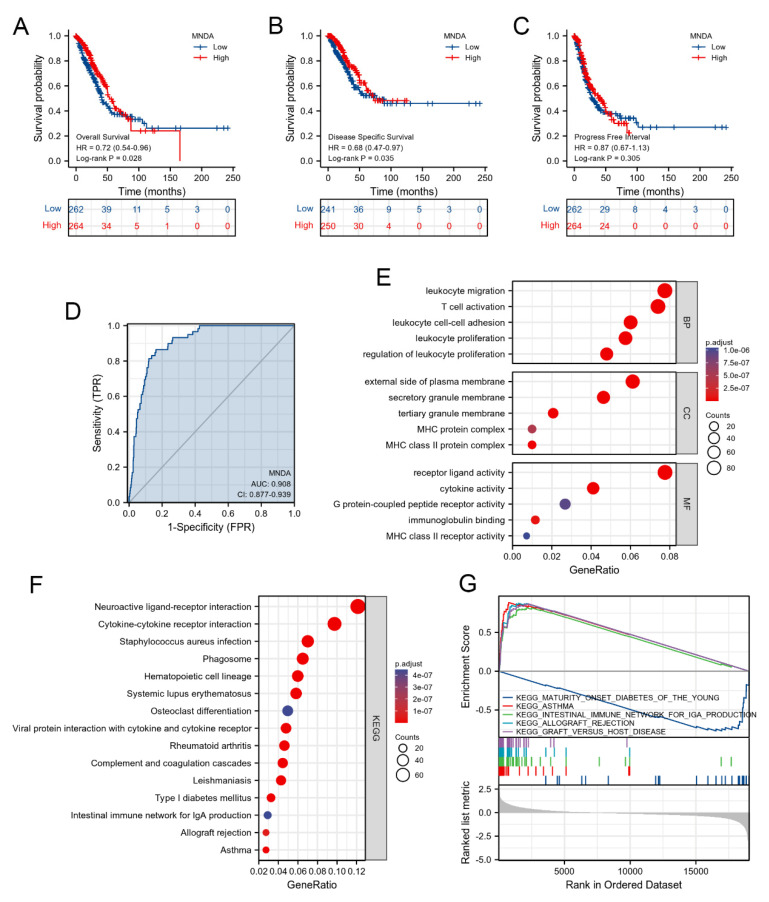
Prognosis and functional analysis of MNDA. (**A**) The high expression of MNDA was associated with good overall survival rate. (**B**) The high expression of MNDA was associated with good disease-specific survival. (**C**) The prognostic effect of MNDA on progress-free interval was analyzed. (**D**) ROC analysis evaluated the value of MNDA as an indicator for distinguishing normal lung tissue samples from LA samples. (**E**) GO analysis enriched BP, CC and MF involved in significantly different genes between MNDA high and low expression groups in LA. (**F**) KEGG analysis enriched signaling pathways between MNDA high and low expression groups in LA. (**G**) GSEA was performed to determine differences in signaling pathways between low- and high-expressing MNDA groups in LA.

Prognostic value is an important indicator for evaluating biomarkers. We used the log-rank method to evaluate the prognostic role of MNDA in overall survival (OS), disease-specific survival (DSS), and progression-free interval (PFI) in LA. The results showed that MNDA was related to OS (HR = 0.72 (0.54–0.96), *p* = 0.028) and DSS (HR = 0.68 (0.47–0.97), *p* = 0.035), but not to PFI (HR = 0.87 (0.67–1.13), *p* = 0.205), which suggested that high expression of MNDA was associated with good prognosis of LA (Figure 2A–C). MNDA can also be used as an indicator to distinguish LA samples from normal lung tissue. The results of ROC curve analysis showed that the AUC value of MNDA was 0.908, and the 95% confidence interval (95% CI) = 0.877–0.939 (Figure 2D).

Although some studies on the function of MNDA have been published, the role of targeting MNDA in LA is still unknown. GO and KEGG analysis are effective means for functional enrichment analysis of genes. Therefore, we divided the samples in TCGA-LUAD into the high and low groups according to the expression of MNDA and screened significantly differential genes with *p* < 0.05 and log2 ≥ |1| as the screening criteria. We obtained a total of 1278 significantly different genes, of which 541 were significantly up-regulated and 737 were significantly down-regulated. After GO analysis of the significantly different genes, it was found that biological process mainly involved leukocyte proliferation, migration, adhesion and T cell activation. Molecular function mainly involved receptor ligand activity, cytokine activity, immunoglobulin binding, G protein-coupled peptide receptor activity and MHC class II receptor activity. In addition, cellular component mainly involved external side of plasma membrane, secretory granule membrane, tertiary granule membrane, and MHC protein complex (Figure 2E). KEGG analysis of significantly different genes showed that signaling pathways were mainly enriched in neuroactive ligand–receptor interaction, cytokine–cytokine receptor interaction, phagosome, intestinal immune network for IgA production, type I diabetes mellitus, asthma, etc. (Figure 2F). Gene set enrichment analysis (GSEA) was further performed to analyze the function of MNDA in LA. As shown in Figure 2G, we found that Maturity onset diabetes of the young, Asthma, Intestinal immune network for lgA production, Allograft rejection and Graft versus host disease were enriched in MNDA high expression phenotype. This suggests that the function of MNDA is mainly involved in the activation of immune cells. The gene lists used for GO, KEGG and GSEA analysis are shown in Supplementary Table S1.

### 4.3. MNDA Is Related to Immune Cell Infiltration of LA

**Figure 3:**
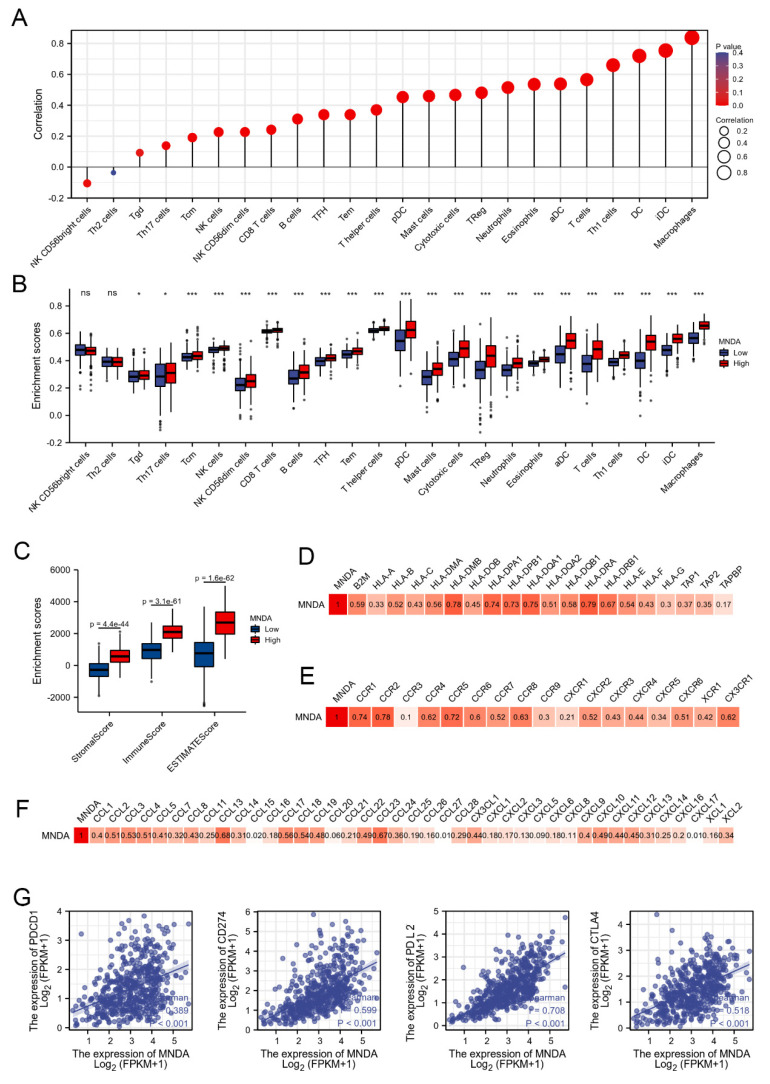
MNDA chemotaxis activates immune cells. (**A**) Correlation analysis between MNDA and 24 kinds of immune cells by ssGSEA. (**B**) Comparison of enrichment scores between high and low expression of MNDA in 24 immune cells in LA. (**C**) Comparison of enrichment scores of StromalScore, ImmuneScore and ESTIMATEScore between high and low MNDA expression. (**D**) Correlation analysis between MNDA and MHC molecules. (**E**) Correlation analysis between MNDA and chemokine receptors. (**F**) Correlation analysis between MNDA and chemokines. (**G**) Correlation analysis between MNDA and immune checkpoints (* *p* < 0.05 and *** *p* < 0.001 ).

To verify the role of MNDA in the activation of immune cells, we used the ssGSEA algorithm in the GSVA package [18,22] to analyze the correlation between MNDA and 24 immune cells, and the results showed that the correlation coefficient of MNDA and macrophages was the highest, which is consistent with the immunohistochemical results for LA in the human ALTAS database (Figure 3A). In addition, ssGSEA analysis also showed that aDC (activated DC); B cells; CD8 T cells; cytotoxic cells; DC; eosinophils; iDC (immature DC); macrophages; mast cells; neutrophils; NK CD56dim cells; NK cells; pDC (plasmacytoid DC); T cells; T helper cells; Tcm (T central memory); Tem (T effector memory); Tfh (T follicular helper); Tgd (T gamma delta); Th1 cells; Th17 cells; and Treg had higher enrichment scores in the MNDA high expression group (Figure 3B). Similarly, StromalScore, ImmuneScore and ESTIMATEScore also had higher enrichment scores in the MNDA high expression group (Figure 3C). We then analyzed the correlation of MNDA with chemokines, chemokine receptors, and MHC molecules. The results showed that MNDA was significantly correlated with chemokines, chemokine receptors, and MHC molecules, which confirmed the results of GO analysis and KEGG analysis (Figure 3D, E, F). Unexpectedly, however, immune checkpoint genes were also significantly positively correlated with MNDA, which is contrary to the function of MNDA that may activate immunity (Figure 3G). The reason for this result may be that MNDA and immune checkpoint genes have a certain cooperative expression pattern.

### 4.4. Abnormal DNA Methylation Level of MNDA in LA

**Figure 4:**
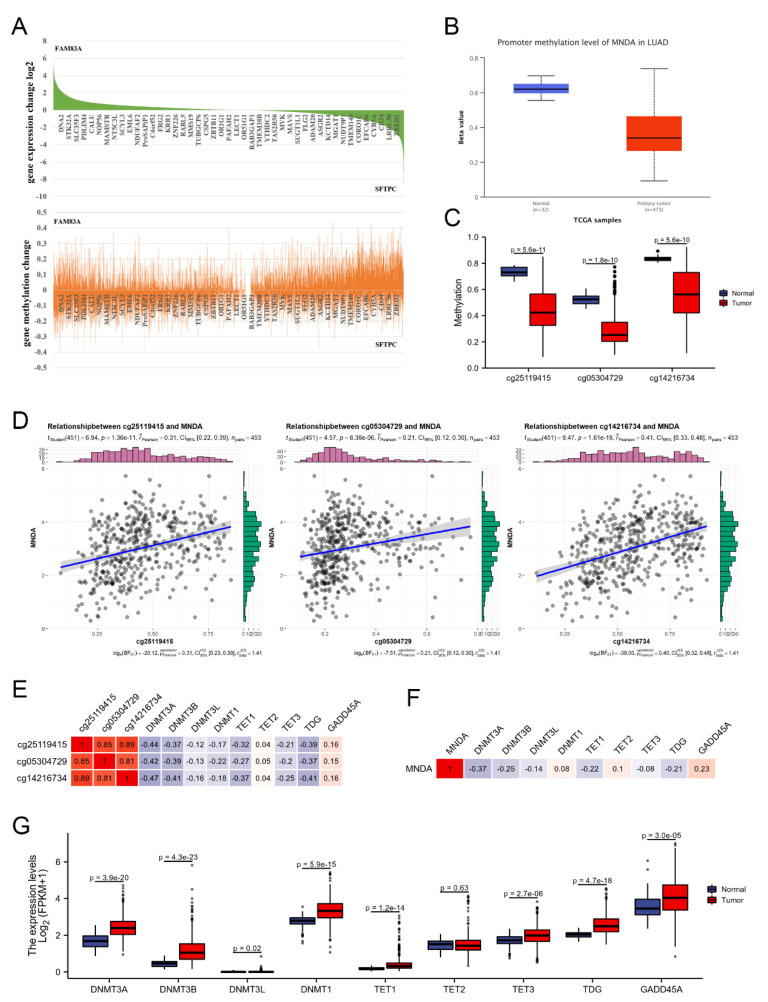
Abnormal DNA methylation level of MNDA in LA. (**A**) Ranking analysis of significantly differential genes and methylation change of significantly differential genes in LA. (**B**) Comparison of promoter methylation levels of MNDA between tumor and normal samples. (**C**) Comparison of methylation levels at MNDA methylation sites between LA and normal samples. (**D**) Correlation analysis of MNDA methylation sites cg05304729, cg25119415, and cg14216734 with MNDA mRNA expression. (**E**) Correlation analysis of DNA methylation and demethylation regulators with MNDA methylation sites cg05304729, cg25119415, and cg14216734 in LA. (**F**) Correlation analysis between DNA methylation and demethylation regulators and MNDA in LA. (**G**) Comparison of DNA methylation and demethylation modulators between LA and normal samples.

DNA methylation and demethylation are important epigenetic mechanisms that have been shown to regulate life processes such as cell proliferation, differentiation, and apoptosis through the activation or repression of many genes [23]. DNA methylation and demethylation are a dynamic equilibrium process, and abnormalities of this process can lead to the occurrence of LA [24]. Numerous studies have shown evidence linking DNA hypermethylation with gene repression, while hypomethylation or demethylation is associated with gene activation [23]. It is very interesting that when we analyzed the correlation between gene expression levels and changes in DNA methylation levels, we found that changes in DNA methylation levels did not always show a negative correlation with changes in gene expression levels (Figure 4A). We then assessed the methylation level of MNDA in the promoter region using the UALCAN database, and the results showed that the promoter methylation level of MNDA in LA was significantly lower than that in normal lung samples (Figure 4B). The methylation levels of three CpG methylation sites of MNDA were also significantly down-regulated in LA (Figure 4C). We performed a correlation analysis between the methylation level and expression level of MNDA, and the results showed that there was a positive correlation between them. The correlation coefficient between cg05304729 and MNDA was Rpearson = 0.21, *p* = 6.38 × 10^−6^, between cg25119415 and MNDA it was Rpearson = 0.31, *p* = 1.38 × 10^−11^, and between cg14216734 and MNDA it was Rpearson =0.41, *p* = 1.61 × 10^−19^ (Figure 4D). This is contrary to the opinion that DNA hypermethylation is associated with gene repression, while hypomethylation or demethylation is associated with gene activation. DNA methylation is reversible because of the presence of methylases and demethylases in living organisms.

The balance between methylation and demethylation maintains the normal spatiotemporal specific expression of many genes. Therefore, we analyzed the correlation of these factors with the expression level and the methylation level of MNDA. The results showed that DNMT3A, DNMT3B, TET1, TET3, and TDG1 were significantly negatively correlated with cg05304729, cg25119415, and cg14216734, respectively, and also showed a significant positive correlation with the expression of MNDA. However, in contrast, GADD45A showed a significant positive correlation with cg05304729, cg25119415, and cg14216734, and also showed a significant positive correlation with the expression of MNDA (Figure 4E,F). In addition, we also found that methylase DNMT3A, DNMT3B, DNMT3L, DNMT1 and demethylase TET1, TET3, TDG1, and GADD45A were all significantly up-regulated in LA, excluding TET2 (Figure 4G), indicating that there may be an imbalance between methylation and demethylation in the process of MNDA methylation in LA.

### 4.5. Transcription Factor SPI1 Regulates the Expression of MNDA 

**Figure 5:**
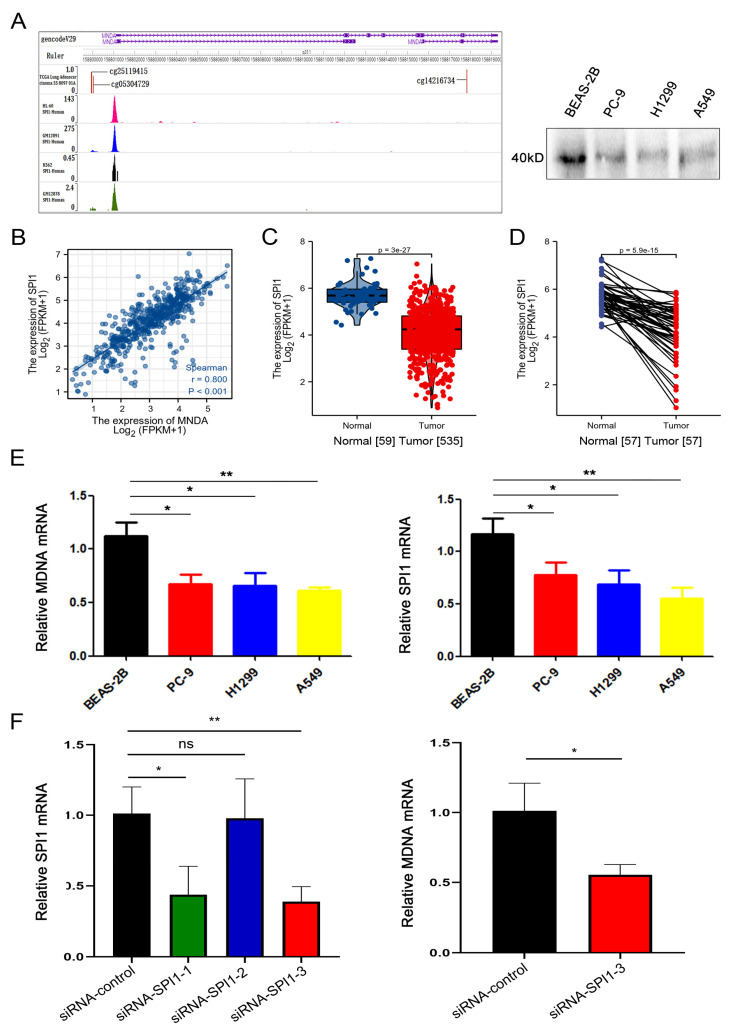
Transcription factor SPI1 regulates the expression of MNDA. (**A**) SPI1 has obvious binding capacity near the transcription initiation site of MNDA. (**B**) Positive correlation between SPI1 and MNDA mRNA expression. (**C**) Unpaired analysis showed that the mRNA expression level of SPI1 in LA was significantly lower than that in normal tissues (normal = 59, tumor = 535, *p* = 3 × 10^−27^). (**D**) Paired analysis showed that the mRNA expression level of MNDA in LA was significantly lower than that in normal tissues (normal = 57, tumor = 57). (**E**) qPCR detection of MNDA and SPI1 in LA cell lines H1299, PC9 and A549, and compared with normal lung cell line Beas-2b. (**F**) qPCR detection of MNDA and SPI1 after down-regulation of SPI1 (* *p* < 0.05 and ** *p* < 0.01, ).

The results we obtained regarding the relationship between the expression of MNDA and its methylation were confusing. Therefore, in order to explore the possible mechanism of the abnormal pattern in the expression of MNDA, we used enrichr database and found that SPI1 was the upstream transcription factor of MNDA, and used the online tool The WashU Epigenome Browser to find that SPI1 binds near the transcription initiation site of MNDA, and SPI1 has a significant binding capacity near the transcription initiation sites of MNDA in Hl-60, K562, GM12891 and GM12878 cell lines (Figure 5A), which was confirmed by in vitro binding experiments (Figure 5A). Moreover, an obvious positive correlation existed between SPI1 and MNDA mRNA expression (r = 0.800, *p* < 0.001) (Figure 5B). In addition to lung cancer, other types of tumors were also consistent (Data not shown). 

We then examined the expression of SPI1 in LA and found that SPI1 was significantly down-regulated in tumor samples (normal 59, cancer 535, *p* = 3 × 10^−27^) (Figure 5C). High expression of SPI1 was associated with good OS (*p* value = 0.03611, log-rank test 4.392, high 251, low 251) (Figure 5D), so we hypothesized that the down-regulation of MNDA expression might be associated with the down-regulation of SPI1 in LA.

To further confirm this conclusion, we used qPCR to detect the mRNA expression of MNDA in LA cell lines H1299, PC9 and A549, as well as in normal cell line Beas-2b. The results showed that the expression levels of MNDA in H1299, PC9 and A549 were significantly lower than that in Beas-2b. Additionally, the expression trend of SPI1 was consistent with that of MNDA (Figure 5E). Next, we designed siRNA to block the expression of SPI1. The results showed that MNDA expression was reduced after down-regulation of SPI1 expression (Figure 5F).

### 4.6. miRNAs Inhibit the Expression of MNDA in LA

**Figure 6:**
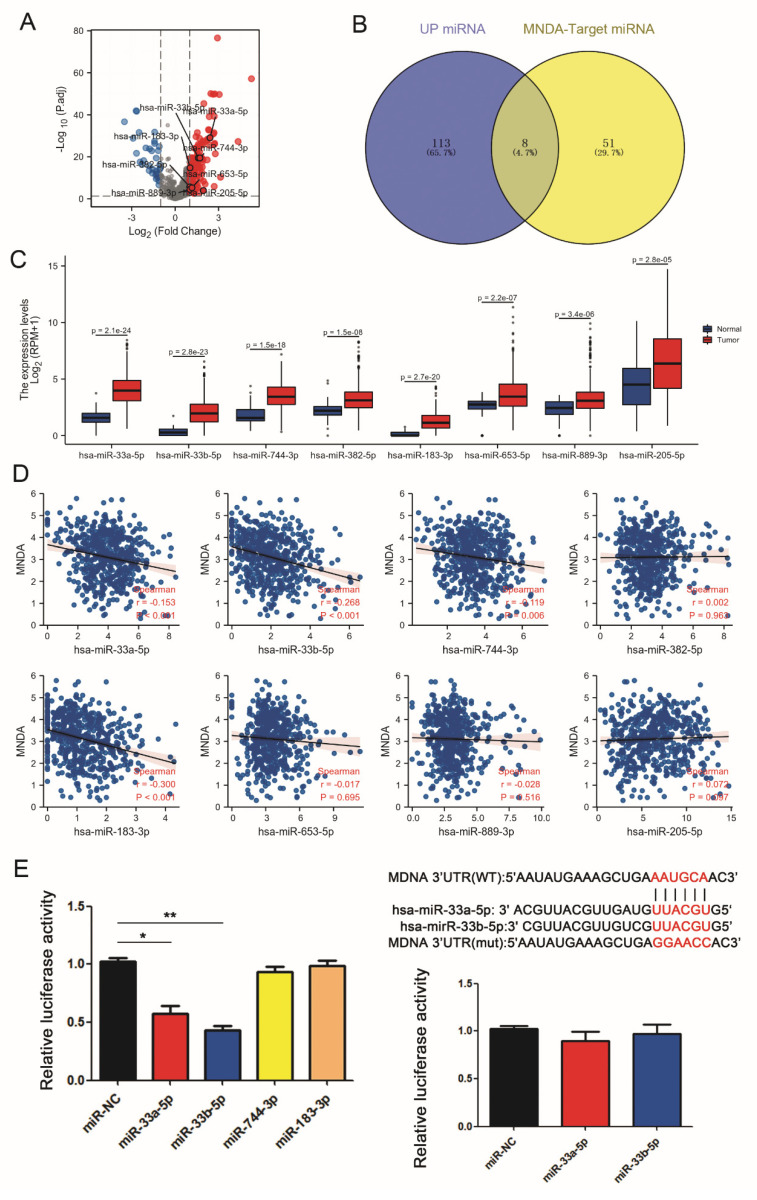
The target miRNAs of MNDA were significantly up-regulated in LA. (**A**) Volcano plot of miRNA expression changes in TCGA-LUAD. (**B**). Wayne analysis of significantly different miRNAs in TCGA-LUAD versus target miRNAs predicted by MNDA. (**C**) The expression of the eight miRNAs in LA. (**D**) Correlation analysis of MNDA with eight target miRNAs. (**E**) Luciferase reporter assay was performed to validate relationship between MNDA and miRNAs (*n* = 4). hsa-miR-33a-5p and hsa-miR-33b-5p strikingly suppressed the luciferase activities of the MNDA-WT reporter vector. The inhibitions of hsa-miR-33a-5p and hsa-miR-33b-5p on luciferase activities were abrogated by the MNDA-MUT reporter vector. ** *p* < 0.01 and * *p* < 0,05 vs. miR-NC.

MicroRNAs (miRNAs) are a class of non-coding single-stranded RNA molecules of approximately 22 nucleotides in length encoded by endogenous genes that bind to the 3′ untranslated region (3′UTR) of mRNAs, resulting in the degradation of target mRNAs and involved in the regulation of gene expression in eukaryotic cells. We speculated that microRNAs are involved in the regulation of MNDA expression in lung cancer. Through RNAseq analysis of miRNA expression in TCGA, we found that 121 miRNAs were up-regulated and 33 miRNAs were down-regulated. We then used Targetscan Human to predict that MNDA has 59 conserved miRNAs (Figure 6A). Intersecting predicted miRNA and differential expressed miRNA, there were eight significant up-regulated miRNAs including hsa-miR-33a-5p, hsa-miR-33b-5p, hsa-miR-744-3p, hsa-miR-382-5p, hsa-miR-183-3p, hsa-miR-653-5p, hsa-miR-889-3 and hsa-miR-205-5p (Figure 6B). The expression of these miRNAs in lung carcinoma is shown in Figure 6C. We next investigated the correlation between the expression levels of the eight miRNAs and MNDA expression in lung carcinoma. The expression levels of hsa-miR-33a-5p, hsa-miR-33b-5p, hsa-miR-744-3p, and hsa-miR-183-3p were negatively correlated with the expression of MNDA (Figure 6D), while the other four miRNAs had no correlation (data not shown).

The luciferase assays were carried out to detect the relationship between MNDA and hsa-miR-33a-5p, hsa-miR-33b-5p, hsa-miR-744-3p, and hsa-miR-183-3p. As illustrated in Figure 6E, over-expression of hsa-miR-33a-5p and hsa-miR-33b-5p strikingly suppressed the luciferase activities of the MNDA-WT reporter vector, which could be reversed by MNDA-MUT reporter vector (Figure 6E). Taken together, our findings deemed that hsa-miR-33a-5p and hsa-miR-33b-5p could inhibit the expression of MNDA through directly targeting it.

## 5. Discussion

Lung cancer is one of the most commonly diagnosed cancers and the leading cause of cancer-related death worldwide, with an estimated 2 million new cases and 1.76 million deaths each year. Over the past two decades, our understanding of lung cancer biology, the use of predictive biomarkers, and improvements in therapy, especially targeted therapy and immune checkpoint inhibitors, have made remarkable progress for improved outcomes [25]. However, there is still a need to find more effective markers to deal with the threat of lung cancer to humans.

MNDA is a nuclear protein and a stress-inducible protein, and reported studies suggest that its function is mainly related to immune cells [26]. MNDA and related proteins contain a pyrin domain that plays a role in signaling related to programmed cell death and inflammation, and studies have shown that MNDA promotes the degradation of the anti-apoptotic factor MCL-1 and the apoptosis of myeloid cells, and is negatively correlated with the amount of anti-apoptotic proteins MCL-1 and BCL-2 in human CLL samples [27]. Reduced levels of MNDA gene transcripts have been detected in familial and sporadic cases of myelodysplastic syndrome (MDS) [28]. These variable patterns of dysregulated MNDA expression may be associated with the variable pathophysiology of MDS. MNDA was mainly expressed in bone marrow, appendix, and spleen in human protein atlas RNA-seq normal tissues, and was also highly expressed in lung tissues [29]. Based on the experimental evidence from high-throughput whole-genome expression, non-equilibrium thermodynamic analysis, nonlinear correlation network, and database mining, it was shown that the down-regulation of MNDA is closely related to metabolic dysfunction in breast cancer [30]. Moreover, MNDA has become a potential therapeutic target for sepsis and inflammatory diseases by promoting the apoptosis of inflammatory cells [31]. MNDA can also inhibit the proliferation and migration of osteosarcoma and promote the apoptosis of osteosarcoma cells [32]. However, the role of MNDA in LA remains unclear.

We compared the mRNA expression of MNDA between LA and normal samples in TCGA using paired and unpaired methods, and found that tumor samples had significantly lower MNDA mRNA levels than normal samples, as validated by data from Oncomine. Likewise, data from the UALCAN database and Human ALTAS database confirmed that MNDA protein was also lower in tumor samples. Further analysis showed that MNDA could be used as a prognostic biomarker for OS and DSS in LA, and as an indicator to distinguish tumor samples from normal samples. These results suggest that MNDA can be used as a novel and valuable biomarker for the diagnosis and treatment of LA.

To identify the function of MNDA, we divided LA into high and low expression groups and performed differential gene analysis. The results of GO and KEGG analysis of these differential genes suggested that the functions of MNDA mainly involved leukocyte proliferation and migration, T cell activation and MHC protein complex. Using the ssGESA method, we further validated that MNDA was significantly correlated with chemokines, chemokine receptors, and MHC molecules. These results suggest that MNDA may act as a factor that activates immune infiltration in LA. However, what is puzzling is that MNDA also has a strong correlation with immune checkpoints. We speculate that it may be induced by the cooperative expression pattern between them.

The regulation of gene expression is complex, delicate and multi-level. Gene expression regulation is the molecular basis of cell differentiation, morphogenesis and ontogeny in organisms. DNA methylation is an important part of epigenetics and an important mechanism for the regulation of gene expression. DNA methylation regulates gene expression by recruiting proteins involved in gene repression or by inhibiting the binding of transcription factors to DNA [33]. However, when we analyzed gene expression and gene methylation in LA, we found that the two were not always negatively correlated. For example, the methylation level of the MNDA gene was reduced in the LA samples, and the mRNA expression level was also reduced. Such results are puzzling because they are in contrast to the fact that DNA methylation suppresses gene expression. The reasons for this result are diverse, because DNA methylation is homeostatic, which is influenced by multiple factors that regulate methylation and demethylation. We then found through analysis that methylase DNMT3A, DNMT3B, DNMT3L, and DNMT1 and demethylase TET1, TET3, TDG1, and GADD45A were all significantly up-regulated. This suggests that the hypomethylation of MNDA may be an imbalance between methylation and demethylation, and the specific mechanism needs to be further studied.

To further explore the mechanism of MNDA down-regulation in LA, we predicted that SPI1 is the transcriptional regulator of MNDA, that SPI1 is significantly down-regulated in lung cancer, and that MNDA expression increases with the increase in SPI1 expression. In addition, chipseq data also showed that SPI1 strongly binds to the promoter of MNDA in multiple cell lines. This suggests that the down-regulation of MNDA in LA may be caused by the down-regulation of its upstream transcription factor SPI1, which leads to a decrease in its transcription.

miRNA is involved in many physiological processes such as cell proliferation, differentiation, apoptosis, and tissue and organ formation [34], and plays a vital role in tumorigenesis, cancer progression, and prognosis [31,35,36,37]. Through further analysis, we found that eight miRNAs targeting MNDA were significantly up-regulated in lung cancer. Additionally, hsa-miR-33a-5p and hsa-miR-33b-5p directly targeted the MNDA promoter, which may be the main reason for the down-regulation of MNDA expression in lung cancer. Therefore, miRNAs may also be one of the reasons for the down-regulation of MNDA in LA.

This study has several limitations. First, this experiment mined the expression and prognostic significance of MNDA through online public databases; therefore, further studies with clinical samples are needed to validate these results. Second, we preliminarily explored the immune-related functions of MNDA in LA, and in vitro and in vivo experiments need to be designed to further study the detailed mechanism of MNDA involved in tumor immunity in lung cancer. Third, although we predicted the mechanism of MNDA expression regulation using bioinformatics methods, more powerful evidence to prove the accuracy of our conclusions is still needed.

## 6. Conclusions

MNDA can be used as a novel and valuable biomarker for the diagnosis and treatment of LA. The abnormal expression of MNDA in LA is the result of the combined effects of transcriptional and post-transcriptional regulation.

## Data Availability

The data that support the findings of this study are openly available in the public databases. The corresponding web sites have been provided in the article.

## Supplementary Materials

The following supporting information can be downloaded at: https://www.mdpi.com/article/10.3390/biology11071047/s1, Table S1: Genes list for KEGG&GO analysis.
